# Dataset for electronic and optical properties of Y_2_O_2_S and Er dopped Y_2_O_2_S calculated using density functional theory and simulated x-ray near edge spectra

**DOI:** 10.1016/j.dib.2022.108671

**Published:** 2022-10-22

**Authors:** Nicholas Dimakis, Eric Baldemar Rodriguez, Kofi Nketia Ackaah-Gyasi, Madhab Pokhrel

**Affiliations:** a Department of Physics and Astronomy, University of Texas Rio Grande Valley, Edinburg 78539, USA; b Department of Mechanical Engineering, University of Texas Rio Grande Valley, Edinburg 78539, USA; c Department of Computer Science, University of Texas Rio Grande Valley, Edinburg 78539, USA

**Keywords:** DFT, IPA, RPA, XANES, GW, BSE, Y_2_O_2_S, Er

## Abstract

The computational data presented in this paper refer to the research article “Optical properties and simulated x-ray near edge spectra for Y_2_O_2_S and Er doped Y_2_O_2_S”. We present the data used to calculate the structural, electronic, and optical properties of the Y_2_O_2_S and its Er^+3^ doped counterparts at various concentrations using density functional theory (DFT) and simulated X-ray near edge (XANES) spectra. We report electronic information from DFT and DFT+U generated from the Vienna Ab initio Simulation Package (VASP) using PAW pseudopotentials. We also report VASP calculated optical properties for the host Y_2_O_2_S using the independent particle approximation (IPA), the random phase approximation (RPA), the many-body GW_0_ approximation, and the Bethe-Salpeter equation (BSE) approximation, under the 10-atom unit cell. The IPA calculations are repeated using the 80-atom unit cell for both the host Y_2_O_2_S and the Y_2_O_2_S:Er^+3^ counterparts. The optical properties data include the frequency-dependent real and imaginary parts of the dielectric function, the absorption and extinction coefficients, the refractive index, and the reflectivity. FEFF10 XANES calculations are performed on the Y K-, L_1_-, L_2_-, and L_3_-edges, as well as on the Er M_5_-edge.

**Specifications Table**

**Table:** 

Subject	Physics, Chemistry
Specific subject area	Computational Chemistry, Material Science
Type of data	Table Figure
How the data were acquired	Optimized geometries, electronic structure, and optical properties were calculated using the VASP program. XANES data were calculated using the FEFF10 code.
Data format	Raw Analyzed
Description of data collection	All calculations were performed at the Texas Advanced Computing Center under the Lonestar 6 and Stampede 2 supercomputers using VASP and FEFF 10 codes.
Data source location	• Institution: University of Texas Rio Grande Valley • City/Town/Region: Edinburg, TX • Country: USA
Data accessibility	Repository name: Mendeley Data Data identification number (DOI number): 10.17632/bym3yj3xxc.1 Direct link to the dataset: https://doi.org/10.17632/bym3yj3xxc.1
Related research article	N. Dimakis, E.B. Rodriguez. Jr., K.N. Ackaah-Gyasi, M. Pokhrel, Optical properties and simulated x-ray near edge spectra for Y_2_O_2_S and Er doped Y_2_O_2_S, Materials Today Communications, 33 (2022) 104328 [1]. https://doi.org/10.1016/j.mtcomm.2022.104328

## Value of the Data

• We provide the data for the structural, electronic, and optical properties of Y_2_O_2_S and Y_2_O_2_S:Er^+3^ at various Er concentrations using several computational approaches. These data are useful to experimentalists for predicting the Y_2_O_2_S bandgap and it's change due to Er doping.
• We provide calculated X-ray absorption near edge structure (XANES)  spectra using the FEFF 10 code, which can be used by experimentalists to analyse transitions in the Y and Er X-ray edges. These data also include projected densities of states per orbital, which are used for electron transition assignments at the X-ray pre-edge and edge regions.
• We provide data from different approximations on calculating optical properties, thus showing their accuracy relative to experiments. These data can also be used by computational chemists for further improvement.
  Our electronic calculations show the presence of the partially filled Er-4f band at the Fermi energy, which agrees with the experimentally observed Er f-f intraband transitions.

## 1 Objective

This dataset can be used by computational material scientists to reproduce the electronic and optical properties calculations and explore how the parameters used in the input files affected the calculations’ accuracies for Y_2_O_2_S:Er^+3^ under varying Er concentrations. Moreover, the Vienna Ab initio Simulation Package (VASP) and FEFF 10 input files contain parameters that can be used for similar calculations of other materials. The data reported for optical properties calculations for the host Y_2_O_2_S refer to different approximations, which affect accuracy, when compared with experimental data. The most accurate approaches for optical properties calculations (i.e., GW and BSE) are not always feasible due to the size of the supercell. In this case, our data show that optical properties calculations using the least accurate IPA method provide sufficiently accurate results relative to the more CPU and memory demanding RPA methods, for energies up to 25 eV. Overall, this dataset adds value to the original published article due to the reproducibility of the data.

## 2 Data Description

**Fig. 1:**
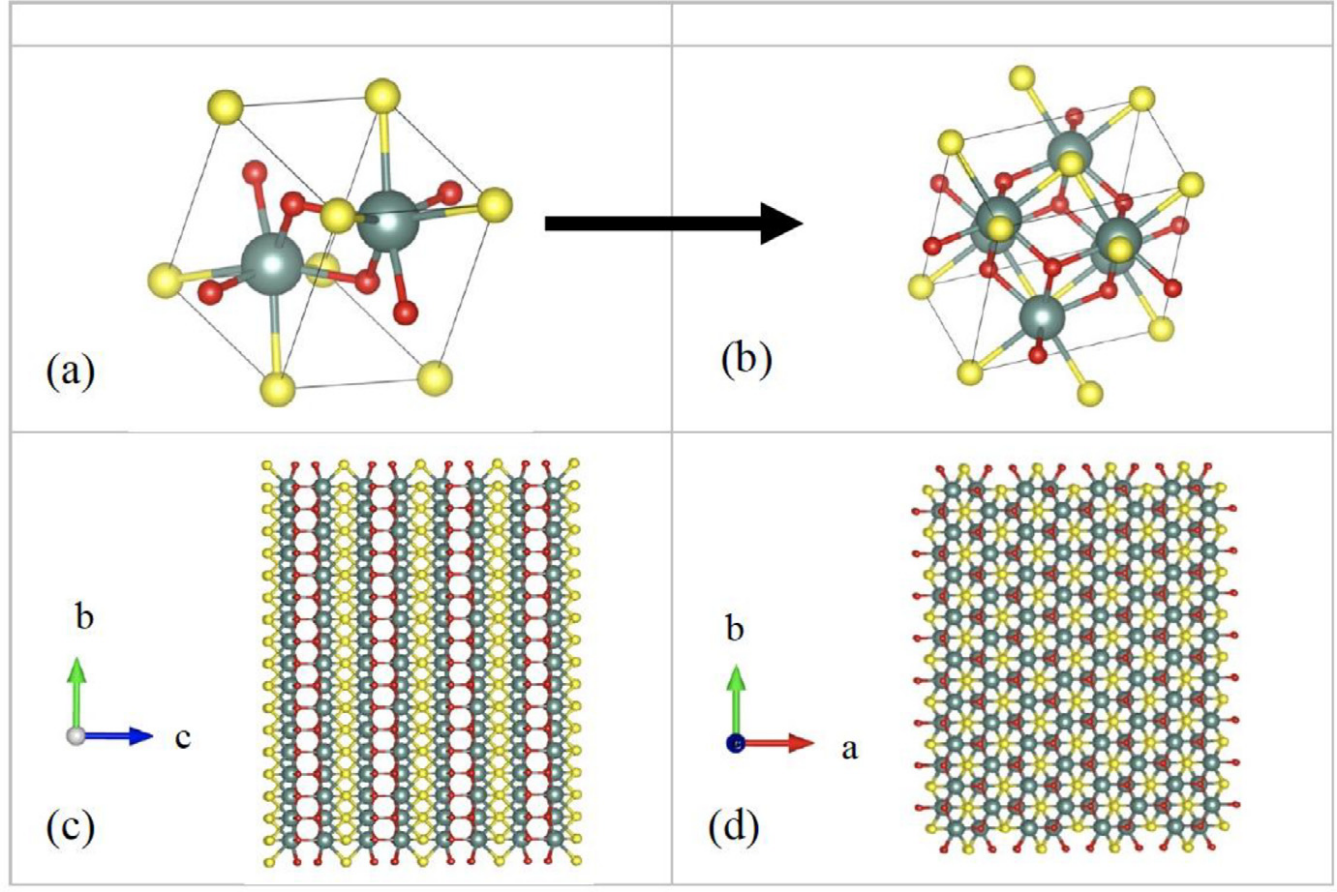
(a) The Y_2_O_2_S unit cell in its triclinic form used for the k-space FEFF 10 calculations and (b) in a cubic form after using the transformation of eq. (1) for i = 1, used by DFT (b), and (c) FEFF 10 geometry configurations for Y_2_O_2_S:Er^+3^, with the absorbing Er atom located close to the center of the cuboid. Thin lines denote the unit cell boundaries. Atoms are colors as follows: Y, O, and S are green, red, and yellow, respectively.

Fig. 1 shows the geometrically optimized unit cells used in our periodic density functional theory (DFT) [2] and FEFF 10 calculations, as well as the molecular clusters used in our FEFF 10 [3] calculations for the Y_2_O_2_S:Er^+3^ under 3.125%, 6.25%, and 9.175% concentrations. The DFT calculations are performed using the 10-atom and 80-atom unit cell. The FEFF 10 calculations for the Y_2_O_2_S:Er^+3^ require large clusters, where the absorbing atom is close to the center of the cluster. For the host Y_2_O_2_S, the FEFF calculations use a periodic k-space approach [4]. DFT and DFT+U [5] are used to calculate the Y_2_O_2_S and the Y_2_O_2_S:Er^+3^ bandgaps, whereas the many-body GW_0_ approximation is also used for the bandgap of the host Y_2_O_2_S:Er^+3^ (Table 1). DFT underestimates the bandgaps relative to experiments, whereas DFT+U improves the bandgap value. However, this improvement is fortuitous, since DFT+U alters the conduction band and changes the bandgap from indirect to direct, in contrast with the experiments. The GW_0_ overestimates the bandgap, whereas the best agreement is obtained from the BSE approximation.

**Table 1:** Bandgaps (E_g_) for the Y_2_O_2_S and its Er^+3^doped counterparts per method used.

Y_2_O_2_S		Method		
	DFT	DFT+U	GW_0_+IPA^b^	GW_0_+BSE
	E_g_ (eV)			
Y_2_O_2_S	3.01	3.80^a^	5.30	5.08
Y_2_O_2_S: 3.125 % Er^+3^	2.73, 0.13	2.92^a^, 0.13^a^		
6.25 % Er^+3^	2.75, 0.18	2.96^a^, 0.34^a^		
9.175 % Er^+3^	2.77, 0.20	2.94^a^, 0.38^a^		

a DFT+U data with U_d_(Y) =12 eV and J_d_(Y) = 1 eV.

b Same as GW_0_+RPA^b^.

**Fig. 2:**
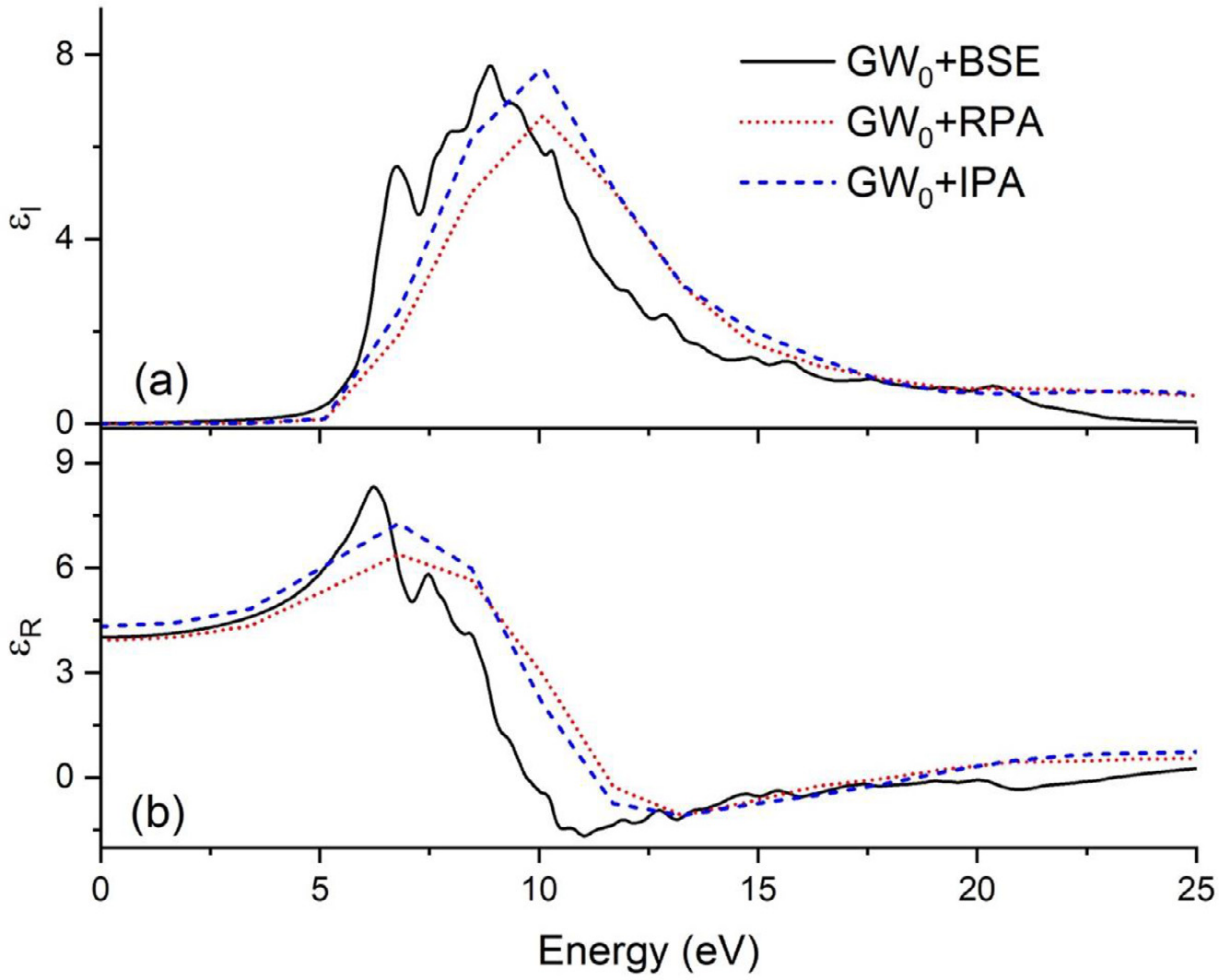
(a) The imaginary part of the frequency-dependent dielectric function (ε_I_) using GW_0_ under the IPA, RPA, and BSE and (b) the real part (ε_R_).

Fig. 2 shows the frequency-dependent dielectric function using GW_0_ [6, 7] under the independent particle method (IPA) [8, 9], the random phase approximation (RPA) [10], and the BSE [11] approximation. The BSE provides accurate bandgap and excitonic information. The BSE and the GW_0_ show similar values for the static dielectric constant ε_R_(0), whereas the best agreement with experiments is provided by the GW_0_ under the IPA.

**Fig 3:**
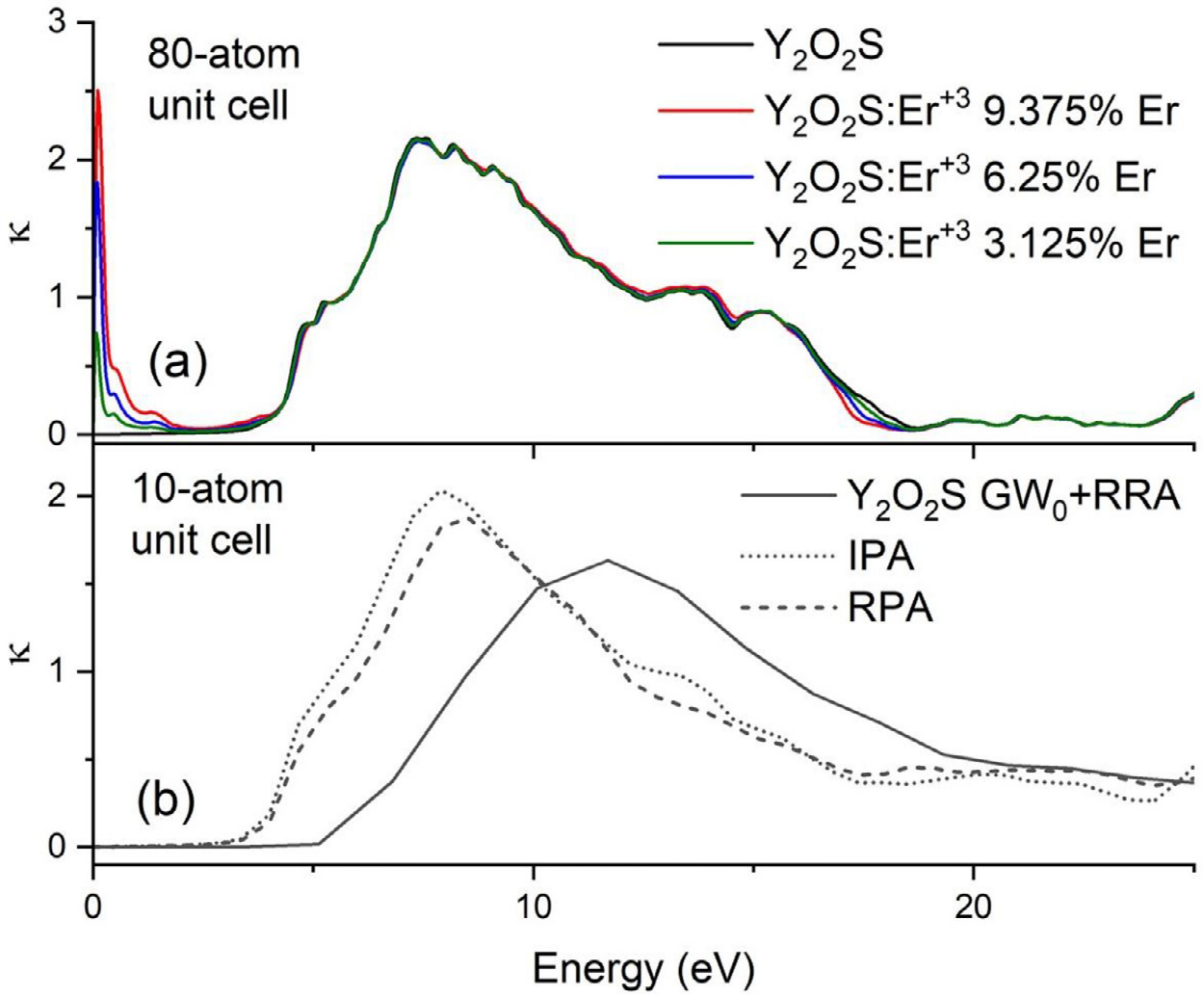
The frequency-dependent extinction coefficient κ(ω) for (a) the undoped Y_2_O_2_S and its Er^+3^ doped counterparts using the IPA and the 80-atom unit cell and (b) the undoped Y_2_O_2_S using the 10-atom unit cell under the IPA, RPA, and GW_0_+RPA.

Fig. 3 shows the frequency-dependent extinction coefficient κ(ω) for the host Y_2_O_2_S and its Er^+3^ doped counterparts under the 80-atom cell. The GW_0_ calculations show a shift on the κ(ω) onsite, in agreement with the larger bandgap predicted by GW_0_ relative to DFT. The κ(ω) spectrum for the Y_2_O_2_S:Er^+3^ at the energies below the bandgap show several peaks, which correspond to the Er f-f intraband transitions.

Table 2 shows the static refractive index and reflectivity, as well as the maximum of the refractive index, the reflectivity, and the absorption coefficient, for the host Y_2_O_2_S and its Er doped counterparts. The energy locations for the above maximum values are also given. The denser 8×8×8 Brillouin zone (BZ) grid does not significantly change the optical properties of the host Y_2_O_2_S. Increased Er doping increases the static refractive index and reflectivity. For the host Y_2_O_2_S, the static values of the refractive index and reflectivity are larger for the IPA and lower for the GW_0_+RPA. Table 3 shows the FEFF calculated X-ray edges of Y and Er, which are compared with experimental data. A good agreement between the calculated and the experimentally reported values is obtained for the Y L_2_- and L_3_- edges and the Er L_3_-edge.

**Table 2:** The static refractive index n(0) and reflectivity R(0), the maximum values of the refractive index $n{\left(\omega \right)}_{\text{max}}$, reflectivity $R{\left(\omega \right)}_{\text{max}}$, and absorption coefficient $\alpha {\left(\omega \right)}_{\text{max}}$ and their locations in the energy spectra per method and unit cell configuration for the host Y_2_O_2_S and its doped counterparts. The energy locations ω are given in parentheses. Values in square brackets refer to the calculations using the Γ-centered 8×8×8 grid, whereas all other values were obtained using the 6×6×6 grid.

	Material & Method					
	Y_2_O_2_S			Y_2_O_2_S:Er^+3^		
				3.125% Er	6.25% Er	9.375 % Er
Property	IPA	RPA	GW_0_+RPA	IPA^2^		
n(0)	2.31 [2.33] 1	2.17 [2.19]	1.98			
	2.28^2^			3.43	5.17	6.16
$n{\left(\omega \right)}_{\text{max}}$	3.11 [3.06] 1	2.86 [2.85]	2.58			
(ω)	4.68 [4.68] 1		8.49			
$n{\left(\omega \right)}_{\text{max}}$	3.24^2^			3.21	3.19	3.17
(ω)	4.52^2^			4.54	4.59	4.64
R(0)	15.89 [16.03] 1	13.96 [13.96]	10.87			
	15.42^2^			30.34	36.04	51.45
$R{\left(\omega \right)}_{\text{max}}$	41.17 [39.91]	38.78 [37.20]	34.19			
(ω)	8.5 [8.52]		13.28			
$R{\left(\omega \right)}_{\text{max}}$	45.84^2^			45.35	45.03	44.93
(ω)	9.57^2^			9.59	9.60	9.66
$\alpha {\left(\omega \right)}_{\text{max}}$	1.70 [1.68]	1.66 [1.62]	1.944			
(ω)	8.50 [8.52]		11.66			
$\alpha {\left(\omega \right)}_{\text{max}}$	1.80			1.74	1.69	1.75
(ω)	9.07			8.26	7.44	8.30

1 10 atom unit cell.

2 80 atom unit cell.

**Table 3:** X-ray Y and Er edges as calculated by FEFF 10 and compared with experimental data.

Element	X-ray edge	Experimental	FEFF
Y	K	17038.4	17059.95
	L_1_	2372.5	2387.81
	L_2_	2155.5	2157.40
	L_3_	2080.0	2082.14
Er	L_3_	8357.9	8354.04
	M_5_	1409.0	1425.33

The submitted data are grouped in four directories. Three of these four directories contain data which refer to the doped Y_2_O_2_S:Er^+3^ compounds at 3.125%, 6.25%, and 9.175% and Er concentrations and are named “Y_2_O_2_S-xEr”, where x is 3, 6, and 9. The fourth directory contains data related to the host Y_2_O_2_S. Each of the Y_2_O_2_S-xEr directories contain three subdirectories, where files from FEFF, band structure, and optical properties calculations reside. Moreover, each of the last two directories contain DFT and DFT+U VASP input files and data files that were used for plotting Fig. 3a. The host Y_2_O_2_S contains three subdirectories, two that contain DFT VASP input files and data files used to plot Fig. 2 and Fig 3b. These subdirectories are named using the number of atoms in unit cell (i.e., 10- and 80-atom). The last subdirectory contains the host Y_2_O_2_S FEFF files.

## 3 Computational Design, Materials and Methods

### 3.1 Unit cell modeling

The Y_2_O_2_S unit cell in a triclinic form (space group P$\bar{3}$m1) is used to build 10- and 80-atom supercells in cubic form for Y_2_O_2_S doped with Er^+3^ by using the following transformation matrix

(1) $$T=x\cdot \left(\begin{array}{ccc} 1 & 1 & 0\\ -1 & 1 & 0\\ 0 & 0 & 1 \end{array}\right){|}_{x=1,2}$$

The unit cell has 5 unique atoms as follows: 2 Y, 2 O, and 1 S atom. The XANES spectra for the Y_2_O_2_S: Er^+3^ cases are calculated using 140 atoms clusters of radii ∼ 7.9 Å around the absorbing Er atom.

### 3.2 DFT parameters

We use the periodic DFT code VASP [12], [13], [14], [15] to calculate electronic and optical properties of Y_2_O_2_S and Y_2_O_2_S doped with Er^+3^ at 3.175 %, 6.25 %, and 9.375 % Er^+3^ concentrations. The VASP PAW pseudopotentials were used [16, 17] with Y, O, S, and Er valance electron configurations as 4s^2^4p^6^5s^1^4d^2^, 2s^2^2p^4^, 3s^2^3p^4^, and 5s^2^5p^6^4f^11^5d^1^6s^2^, respectively. The generalized gradient approximation of the Perdew–Burke–Ernzerhof (PBE) functional [18] was used. The long-range electron correlations responsible for van der Waals interactions were approximated by the Grimme [19] D3 semiempirical correction. We used 525 eV for the kinetic energy cutoff for all our calculations, which exceeds the maximum of the default PAW energy cutoff values. The energy SCF convergence and the geometry thresholds are set to 10^−9^ eV per atom and 10^−4^ eV/Å, respectively. The BZ was sampled using the 4 × 4 × 4 Monkhorst-Pack grid for geometry optimizations and electronic information, whereas for the optical properties we used the Γ-centered 6 × 6 × 6 BZ grid. The electronic band structure and the densities of states (DOS) are calculated using two district methods: 1) DFT and 2) DFT+U, where U is the Hubbard U correction parameter by Liechtenstein et. al [5]. The optical properties for the host Y_2_O_2_S are calculated using the IPA, the RPA, the many-body GW_0_ methods as a correction to IPA and RPA (i.e., GW_0_+IPA and GW_0_+RPA), and the BSE approximation [11], the last four using the 10-atom unit cell. The Y_2_O_2_S:Er^+3^ optical properties were calculated using the IPA method, under the 80-atom unit cell.

### 3.3 FEFF 10 parameters

The FEFF10 code [3] is used to calculate XANES through real space Green functions. The atomic potentials have been calculated self-consistently. We include full multiple scattering in all FEFF 10 calculations. The Hedin-Lundqvist pseudopotential [20] was used for the exchange interaction, whereas the absorbing atom core hole was treated using the RPA method. FEFF also calculates projected DOS per atomic orbital. We used 0.1 eV half-width as the Lorentzian parameter for the projected DOS calculations.

## Ethics Statements

This work does not require any ethical statement.

## CRediT Author Statement

**Nicholas Dimakis:** Conceptualization, Methodology, Supervision, Validation, Writing – reviewi & editing; **Eric Baldemar Rodriguez Jr.:** Data curation, Validation, Investigation; **Kofi Nketia Ackaah-Gyasi:** Data curation, Validation, Investigation; **Madhab Pokhrel:** Conceptualization, Methodology, Writing – review & editing, Data curation, Validation.

## Declaration of Competing Interest

The authors declare that they have no known competing financial interests or personal relationships that could have appeared to influence the work reported in this paper.
